# Optimizing differentiated treatment models for people living with HIV in urban Zimbabwe: Findings from a mixed methods study

**DOI:** 10.1371/journal.pone.0228148

**Published:** 2020-01-28

**Authors:** Miriam Rabkin, Michael Strauss, Joanne E. Mantell, Munyaradzi Mapingure, Tsitsi B. Masvawure, Matthew R. Lamb, Jennifer M. Zech, Godfrey Musuka, Innocent Chingombe, Martin Msukwa, Rodrigo Boccanera, Clorata Gwanzura, Gavin George, Tsitsi Apollo

**Affiliations:** 1 ICAP at Columbia University, New York, New York, United States of America; 2 Department of Epidemiology, Columbia University Mailman School of Public Health, New York, New York, United States of America; 3 Health Economics and HIV/AIDS Research Division (HEARD), University of KwaZulu Natal, Durban, South Africa; 4 Department of Psychiatry, Division of Gender, Sexuality and Health, Columbia University, New York, New York, United States of America; 5 The New York State Psychiatric Institute, New York, New York, United States of America; 6 ICAP at Columbia University, Harare, Zimbabwe; 7 Department of Sociology and Anthropology, College of the Holy Cross, Worcester, Massachusetts, United States of America; 8 ICAP at Columbia University, Pretoria, South Africa; 9 Health Resources and Services Administration (HRSA), Bethesda, Maryland, United States of America; 10 Ministry of Health and Child Care, HIV/AIDS and STIs Unit, Harare, Zimbabwe; University of the Witwatersrand, SOUTH AFRICA

## Abstract

**Introduction:**

Zimbabwe is scaling up HIV differentiated service delivery (DSD) to improve treatment outcomes and health system efficiencies. Shifting stable patients into less-intensive DSD models is a high priority in order to accommodate the large numbers of newly-diagnosed people living with HIV (PLHIV) needing treatment and to provide healthcare workers with the time and space needed to treat people with advanced HIV disease. DSD is also seen as a way to improve service quality and enhance retention in care. National guidelines support five differentiated antiretroviral treatment models (DART) for stable HIV-positive adults, but little is known about patient preferences, a critical element needed to guide DART scale-up and ensure person-centered care. We designed a mixed-methods study to explore treatment preferences of PLHIV in urban Zimbabwe.

**Methods:**

The study was conducted in Harare, and included 35 health care worker (HCW) key informant interviews (KII); 8 focus group discussions (FGD) with 54 PLHIV; a discrete choice experiment (DCE) in which 500 adult DART-eligible PLHIV selected their preferences for health facility (HF) *vs*. community location, individual *vs*. group meetings, provider cadre and attitude, clinic operation times, visit frequency, visit duration and cost to patient; and a survey with the 500 DCE participants exploring DART knowledge and preferences.

**Results:**

Patient preferences were consistent in the FGDs, DCE and survey. Participants strongly preferred respectful HCWs, HF-based services, individual DART models, and less costly services. Patients also preferred less frequent visits and shorter wait times. They were indifferent to variations in HCW cadre and distances from home to HF. These preferences were mostly homogenous, with only minor differences between male *vs*. female and older *vs*. younger patients. HCWs in the KII correctly characterized facility-based individual models as the one most favored by patients; HCWs also preferred this model, which they felt decongested HFs and reduced their workload.

**Conclusions:**

DART-eligible PLHIV in Harare found it relatively easy to access HFs, and preferred attributes associated with facility-based individual models. Prioritizing these for scale-up in urban areas may be the most efficient way to sustain positive patient outcomes and increase health system performance.

## Introduction

The global scale-up of HIV treatment enabled an estimated 23.3 million people living with HIV to access antiretroviral treatment (ART) by the end of 2018 [1]. To reach global targets, however, millions more must learn their HIV status, link to treatment, and maintain viral suppression. Resource-constrained health systems will need to adapt to accommodate the growing number of people on ART and to deliver high-quality HIV services.

In response, many countries are transforming their approach to HIV service delivery to include “differentiated” HIV treatment models [2,3]. These differentiated ART (DART) approaches tailor services to groups of patients, aiming to improve quality and efficiency by varying service location, frequency, individual *vs*. group appointments, health care worker (HCW) cadre, and the use of peer-led services [4,5]. DART models for stable adults doing well on treatment typically include less-intensive services delivered via facility-based individual models (including less frequent appointments with multi-month prescriptions and/or fast-track refill visits); facility-based group appointments (*e*.*g*., club refills); community-based individual models (*e*.*g*., outreach models with ART pick-up locations in the community); and community-based group models (*e*.*g*., family member refills and community ART refill groups) [6,7].

**Fig 1:**
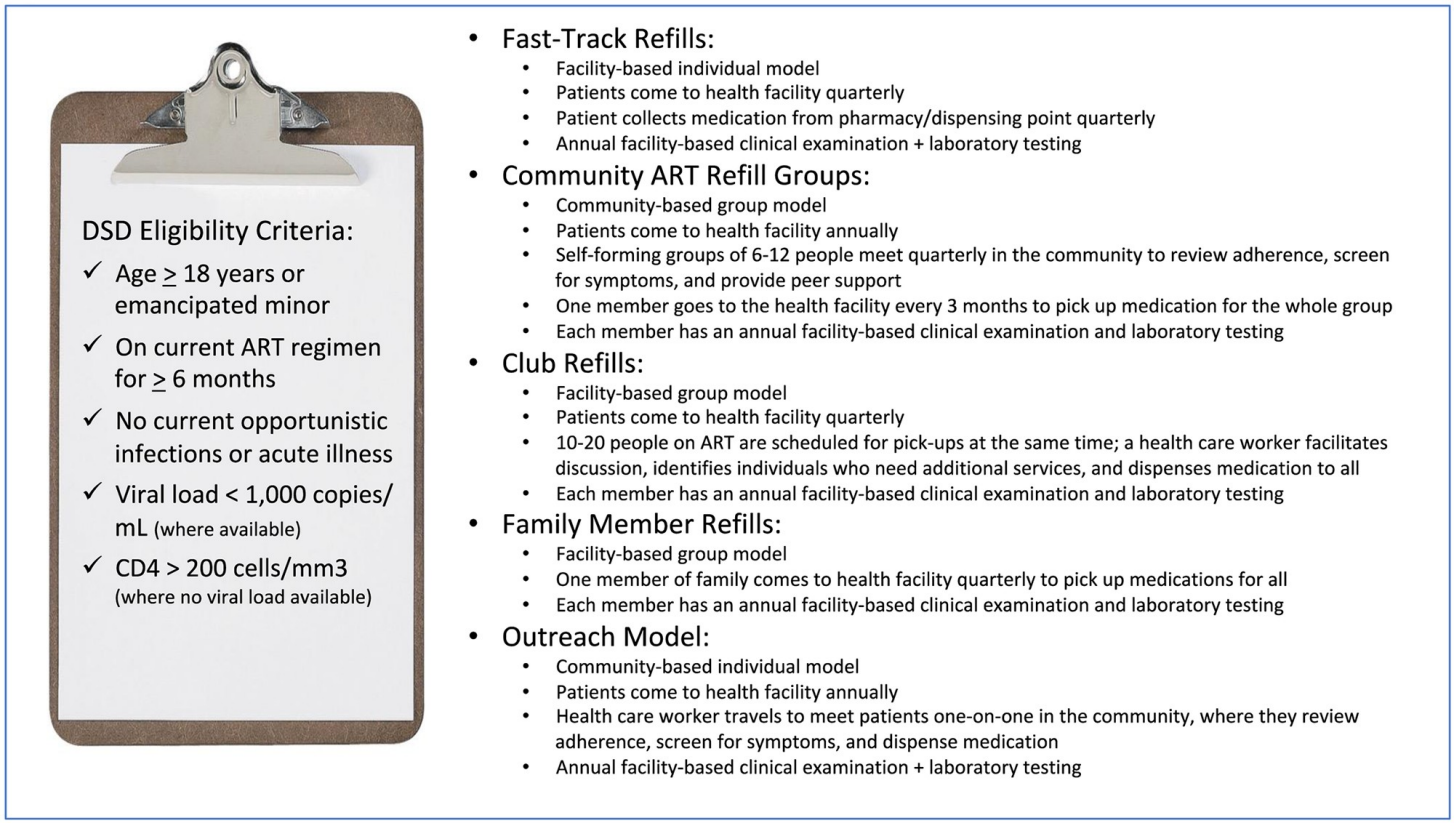
Zimbabwe’s five differentiated service delivery models.

Zimbabwe has a 14.1% adult HIV prevalence, 1.35 million people living with HIV and 1.15 million people on ART. In 2017, the Ministry of Health and Child Care (MoHCC) introduced five DART models (Fig 1) and suggested that health facilities (HFs) implement the combination of models that best fit their contexts and patients [8]. The result has been substantial heterogeneity amongst HFs in terms of the number and type of DART models offered. In November 2018, MoHCC estimated that 35% of eligible patients had been enrolled in DART nationwide and established a target of 65% DART coverage by the end of 2019 [9].

Because DART models are opt-in, understanding patient preferences is critical to program planning [10]. MoHCC program data suggests that DART uptake varies across urban *vs*. rural settings, consistent with studies from other countries [11]. We conducted a mixed methods study in Zimbabwe’s capital city of Harare to explore patient and HCW perspectives with the objective of identifying the optimal characteristics of DART in an urban setting using key informant interviews (KIIs), focus group discussions (FGDs), a discrete choice experiment (DCE), and a patient survey.

## Methods

### Study setting

Data were collected at six purposively-selected public-sector clinics and one public-sector hospital in Harare. Sites were chosen with MoHCC and a local study advisory group; selection criteria included high-volume ART clinics (> 3,000 adults on ART), implementation of at least one DART model, and location. Six of the HFs offered community antiretroviral refill groups (CARGs), six offered Fast Track, four offered the Family ART Group model, and none offered Group Refills or the Outreach Model.

### Study design

Qualitative and quantitative methods were used to explore patient preferences for service delivery characteristics associated with Zimbabwe’s DART models. In Phase One (July 2018), KIIs with HCW and FGDs with DART-eligible patients were conducted to provide contextual information and feedback on the attributes and images used for the DCE. In Phase Two (October-November 2018), a combined DCE and patient survey was conducted to further explore preferred characteristics of DART models. The DCE offered participants a choice between hypothetical scenarios, as described below, and the survey explored participants’ health, social support, satisfaction with current HIV services, experiences with disclosure and stigma, and their knowledge and attitudes regarding DART.

The DCE is a quantitative technique used to elicit participant preferences via hypothetical scenarios [12,13,14]. DCEs enable valuation of individual attributes (*e*.*g*., cost, time) that comprise a scenario (*e*.*g*., a DART model) and thus can be used to design DART models that include the characteristics of highest value. For this study, a literature review, stakeholder discussions, and Phase One KII and FGD data were used to develop the DCE attributes, levels and illustrations used to describe the DART models. Final attributes included: 1) HF *vs*. community location; 2) individual *vs*. group consultations; 3) provider cadre; 4) HF operation times; 5) visit frequency; 6) visit duration; and 7) total visit cost to patient (Table 1). A fractional factorial design was developed with 32 choice sets according to the method described in Burgess and Street (2005) [15] to ensure an optimal design using the D-efficiency criterion. A blocking variable was included in the design to divide the 32 choice sets into four versions so that each participant made 8 choices between two hypothetical DART models illustrated on a “choice card”, with no opt-out option to maximize the amount of information about preference structures. In addition, each of the four versions included a repeat question to check for consistency in the responses, and one question from the first version was included in each of the other versions to ensure consistency in responses across versions. DCE recruitment was stratified by age and gender, with an equal number of younger men, younger women, older men and older women. Age categories of 18–29 years and 30+ years were selected in consultation with local stakeholders.

**Table 1:** Discrete choice experiment attributes and levels.

Attribute	Definition	Level
Location of service delivery	Location/venues where DART services (check up and ART) are provided	Health facility/clinic close to home or workplace (no more than 10 minutes travel time)
		Health facility/clinic further from home or workplace (45 minutes travel)*
		Community-based DART services
		Home-based DART services
Participants/others seen at same visit	Individual appointment vs. an appointment that includes other DART patients or family members	Individual *
		Group
Type of service provider	The person/people who convene the group and deliver services (counseling, weight and vital signs, symptom screening, adherence assessment and/or ART distribution)	Professional health worker who is respectful and understanding
		Professional health worker who is *not* respectful and understanding *
		Peer/lay person who is respectful and understanding
		Peer/lay person who is *not* respectful and understanding
Times (days and hours) of operation	Days and times ART services are provided	Work week only (standard hours: 8am– 4pm) *
		Work week with early morning hours (opens at 5am)
		Work week with evening hours (open until 8pm)
		Work week + weekend hours (7 days a week, 8am-4pm)
Frequency of visits/visit spacing	Frequency of routine visits (e.g., for patients who are feeling well, without new symptoms or concerns that would require a non-routine visit or consultation)	Four times a year (*i*.*e*., every three months) *
		Two times a year (*i*.*e*., every six months)
Total time for visit	Total time for visit, including registration, wait times, and time with providers. Does not include transportation time	30 minutes
		60 minutes *
		2 hours
		4 hours
Total cost of visit	Total cost, including transportation, direct medical costs (e.g., consultation or booking fee, lab costs if not available at public facility, non-ARV drug costs), costs of childcare	Free
		$1
		$3*
		$10

* = reference level, based on typical non-differentiated services

#### Sample size

KII and FGD sample sizes were designed to attain saturation and to enable FGD stratification by age and gender; the goal was a maximum of 35 KIIs and 8 FGDs with 6–8 participants in each. Sample size for the DCE and survey was based on the number of participants needed for the DCE. In DCEs, the optimal number of participants needed for statistical power is determined by the number of choices in each set, the number of attributes, and the number of levels in each attribute. For this DCE, a sample size of 500 was needed to compare the results of four subgroups (younger men/younger women/older men/older women); this was determined by a commonly used rule of thumb per stratification N (125 per stratification), where N ≥ 500L/SJ, for a design in which the maximum number of levels for any attribute L is four, the number of choices in each choice set S is two, and the number of choice sets presented to each participant J is eight [16].

### Recruitment and data collection

#### Key informant interviews

Semi-structured KIIs were conducted with HCWs and lay providers identified by purposive sampling at each HF. Following informed consent, research staff conducted in-person interviews in English; each took approximately 50 minutes. Interviews were audio-recorded, transcribed, and reviewed for accuracy and completeness by senior research staff before analysis.

#### Focus group discussions

At each HF, clinicians and community linkage facilitators identified a convenience sample of adults on ART who were virally suppressed and eligible for DSD but not currently enrolled in DART and referred them to the study team. The team met with potential participants to explain the study, invite them to participate, and obtain informed consent. FGDs were stratified by age and gender, with separate FGDs for younger men and women (18–29 years) and for older men and women (30+ years). FGDs were conducted in Shona (Harare’s most common local language); each took approximately 90 minutes. They were audio-recorded, transcribed and translated into English. A bilingual senior research staff member validated each FGD transcript for completeness and accuracy prior to coding.

#### DCE and patient surveys

Phase Two participants were recruited using the approach and criteria used for FGDs. Participants were stratified by age and gender, with the intent to enroll 50% women and 50% in each age group (18–30, 30+ years). Phase One participants were not eligible for Phase Two. SurveyCTO was used to integrate the DCE and patient survey into a single tablet-based interviewer-assisted survey, which took approximately 45–60 minutes and was conducted in Shona. Data were uploaded for storage on the SurveyCTO cloud-based system, with internal data quality checks for valid entries, skip patterns, range checks, and missing values.

### Data analysis

KIIs and FGDs were entered and analyzed using the Dedoose Software Package (version 8.1.8). A team of two researchers coded each transcript by question and key theme, meeting frequently to compare and reconcile the application of thematic codes. Code reports were analyzed by two researchers who initially worked independently and then met to share and discuss their initial impressions about participants’ views on the different DCE models. These findings were condensed into summaries and shared with other study team members. A team of five researchers conducted framework analysis [17] to organize the data by DART model preference, participants’ positive and negative healthcare experiences, facilitators and barriers to ART adherence, and personal experiences of HIV-related stigma. A final coding scheme was achieved through consensus discussions among the research team [18].

Survey data were transferred from the cloud-based server to the SAS statistical software package (version 9.4), where entries were error-checked and summary variables created to produce a final analytic dataset. Descriptive analyses were conducted using SAS version 9.4. Tests of significance were conducted using Chi-square or Fisher’s Exact tests for categorical variables and t-tests for continuous variables.

DCE analysis was conducted using a fixed effects logit model to estimate the relative utility for each attribute and level. Interaction effects were also estimated for gender and age. The results of the analysis are presented as odds ratios, in relation to a baseline scenario which included the reference levels for each attribute.

### Ethical approvals

The study was approved by Columbia University’s Institutional Review Board (protocol IRB-AAAR9020), the Medical Research Council of Zimbabwe (protocol MRCZ/A/2326), and the U.S Health Resources & Services Administration.

## Results

The study team conducted 35 KIIs with HCWs (five from each site), eight FGDs with 54 participants and 500 surveys including the DCE.

### Participant characteristics

KII participants included 16 nurses and 12 counselors; the remainder were data entry clerks, a nurse’s aide and a community linkage facilitator. Median age was 39 years (range 24–61; IQR 34–47 years) and 24/35 (69%) were female. Twenty-one (60%) had worked at their current HF for over three years. FGD participants were stratified by age and sex, as above. Forty-three of the 54 (80%) had completed at least some secondary education and 35 (65%) were employed either full- or part-time. Survey/DCE participants were also stratified by age and sex, with median age of 29.5 years (IQR: 24–41). Most (91%) had completed secondary education and 51% were employed. Forty percent of survey/DCE participants reported an HIV-positive spouse or partner, and 68% reported an HIV-positive family member. Table 2 summarizes additional information.

**Table 2:** Survey & DCE participant demographics.

		All participants		Male		Female	
		N = 500		N = 250		N = 250	
Age	18–29 years	250	50%	125	50%	125	50%
	30+ years	250	50%	125	50%	125	50%
	Median (IQR)	29.5 years (24–41)		29.5 years (23–42)		29.5 years (24–41)	
Educational attainment	None	1	0%	0	0%	1	0%
	Primary	55	11%	23	11%	27	11%
	Secondary	401	80%	192	77%	209	84%
	> Secondary	43	9%	30	12%	13	5%
Marital status	Single	143	29%	90	36%	53	21%
	Married monogamous	248	50%	119	48%	129	52%
	Married polygamous	9	2%	1	0%	8	3%
	Living with partner	8	2%	3	1%	5	2%
	Divorced/separated	52	10%	28	11%	24	10%
	Widowed	40	8%	9	4%	31	12%
Spouse/partner HIV status amongst those married or living with partner (N = 265)	Positive	191	72%	88	72%	103	73%
	Negative	61	23%	33	27%	28	20%
	Don't know/refused/missing	13	5%	2	2%	11	8%
Children under 18 years in the household	Yes	337	67%	154	62%	183	73%
	No	163	33%	96	38%	67	27%
HIV-positive family member	Yes	339	68%	162	65%	177	71%
	No	137	27%	75	30%	62	25%
	Don’t know	24	5%	13	5%	11	4%
Currently working	Yes	256	51%	159	64%	97	39%
	No	244	49%	91	36%	153	61%
Primary occupation amongst those currently working (N = 256)	Professional	65	25%	48	30%	17	18%
	Self-owned business	89	35%	44	28%	45	46%
	Other business	30	12%	18	11%	12	12%
	Services	50	20%	33	21%	17	18%
	Sex worker	1	0%	0	0%	1	1%
	Other	21	8%	16	10%	5	5%
Income last month amongst those currently working (N = 256)	≤ 100$	87	34%	41	26%	46	47%
	$101-$500	120	47%	85	53%	35	36%
	> 500	44	17%	31	19%	13	13%
	Don’t know/refused/missing	5	2%	2	1%	3	3%

Survey/DCE participants had lived in Harare for a median of 20 years. Half (54%) were married or living with a partner and 51% were currently working, with men more likely to be formally employed than women (64% *vs*. 39%, p < 0.0001). Almost all (93%) had family in the city, 16% reported belonging to a sports or social club and 62% reported attending religious services regularly. The majority of participants reported at least one person with whom they could share their most private concerns and fears (82%), a strong emotional bond with at least one other person (72%), and confidence that if they were sick and needed someone to bring them to the doctor that they could easily find someone to help (75%). While most participants (70%) noted that it was difficult to disclose their HIV status to others, 90% had disclosed their status to at least one person.

### Experience with current ART services

FGD participants had been on ART for a median of seven years (range 0.5–20; IQR 3–9) and reported mixed experiences with HIV treatment. Good quality service, responsive HCWs and availability of medicines were highlighted as positive experiences. Negative experiences included perceptions that privacy was not always protected by HCWs, leading to inadvertent disclosure of HIV status to other clinic attendees. Long wait times at HFs and the costs associated with treatment, such as user fees and transportation costs, were additional challenges.

Nearly half of survey/DCE participants had been on ART for four or more years (median 4.1 years; IQR 1.0–7.6), and most reported high levels of satisfaction with their current HIV treatment. The majority (91%) were “very satisfied” or “somewhat satisfied” with the knowledge and competence of HCWs at their clinic, the respect shown to patients (92%), the communication skills of their current HCW (92%) and their overall HIV care (91%).

The majority of survey participants said it was “easy” or “very easy” for them to get to clinic. Participants typically walked (50%) or took public transportation (44%); 48% said it took them less than 30 minutes and 41% said it took 30–60 minutes. Travel costs varied, with 52% reporting they spent nothing, 22% reporting costs of less than $1USD and 24% spending $1–3 USD. The majority of participants (79%) reported a facility cost (typically a co-payment) of $1–3 USD. Wait times were variable, with 38% of participants reporting spending 1 hour or less at the clinic, 28% reporting 1–2 hours, 24% reporting 2–4 hours and 10% reporting more than a four-hour wait time.

### Participant preferences for DART characteristics and models

**Fig 2:**
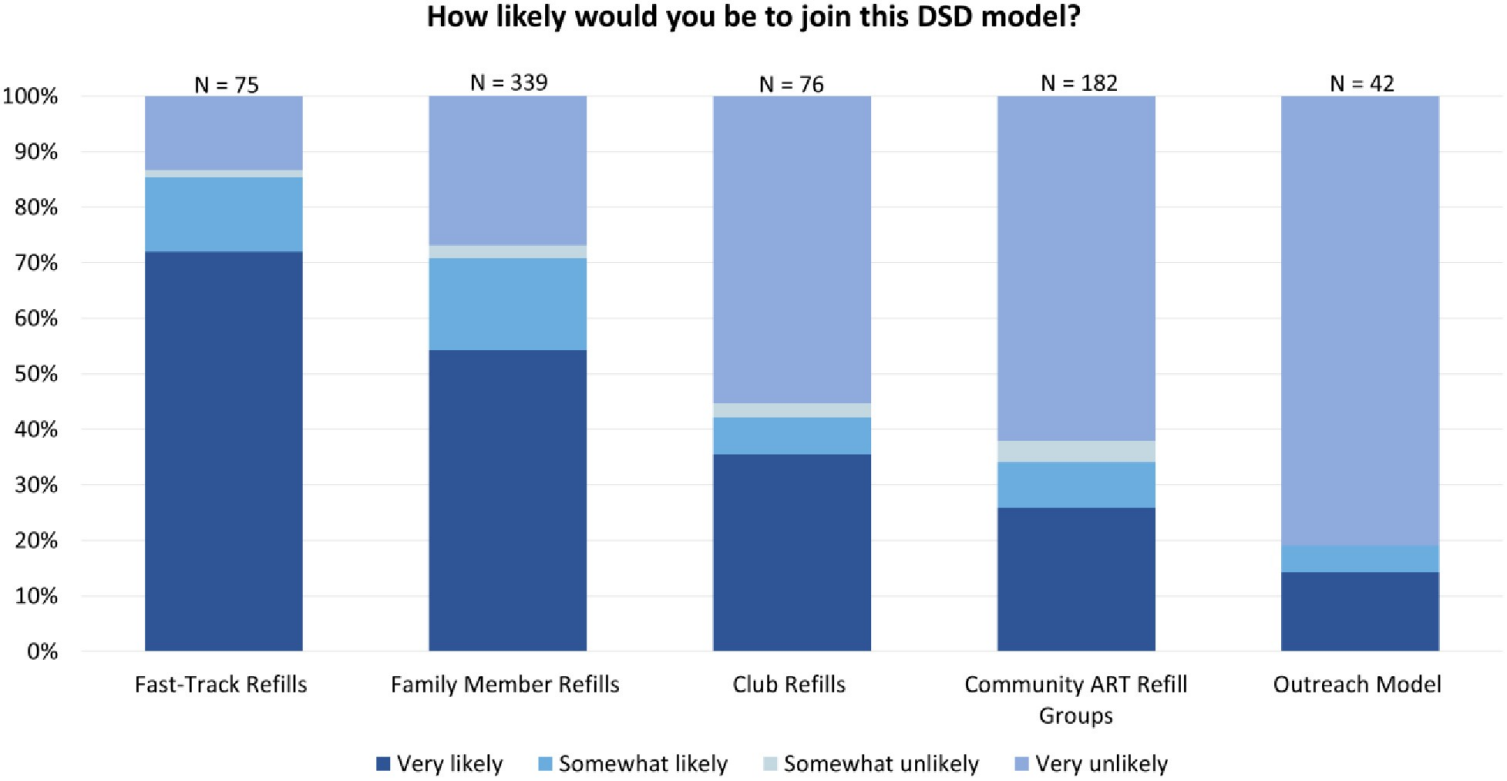
Preferences in patient survey.

**Fig 3:**
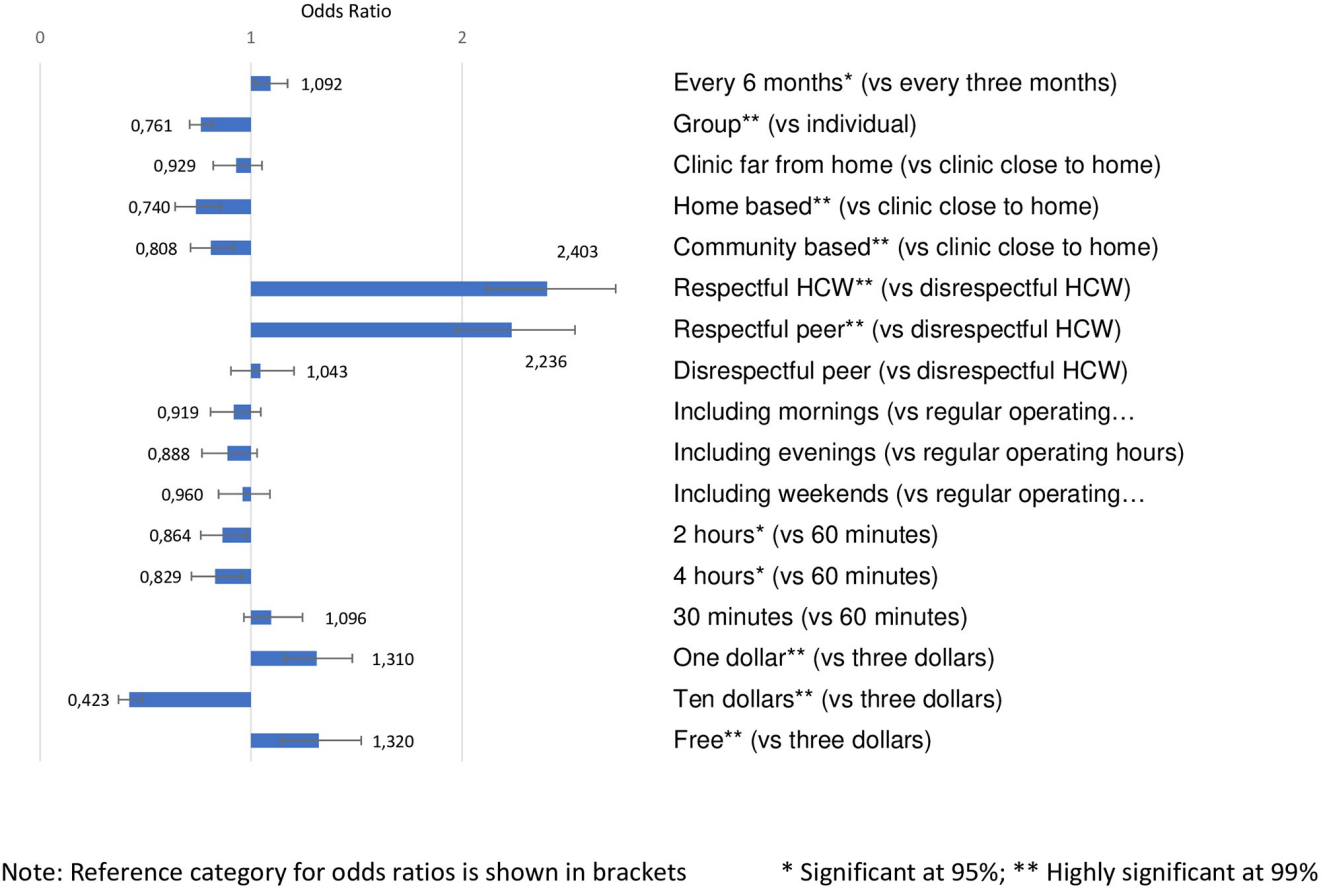
Discrete choice experiment results.

Participant preferences were driven by their desire to interact with respectful HCWs and for efficient ART treatment services, which drove their choices for DART models in the survey (Fig 2) and DART attributes in the DCE (Fig 3).

#### Provider attitude: Respect and confidentiality

Provider attitude was the most valued attribute in the DCE. Participants had no preferences for services delivered by a professional HCW *vs*. a peer/lay provider—the odds ratio for disrespectful peer/lay provider vs disrespectful professional HCW was not significant and the confidence intervals overlap for respectful professional HCWs and respectful peer/lay providers, when compared to disrespectful professional HCWs. However, participants were more than twice as likely to choose services delivered by respectful and understanding staff (OR 2.4, CI 2.11–2.73; p<0.001 for respectful *vs*. not respectful professional HCWs; and OR = 2.1; CI 1.89–2.43; p<0.001for peer/lay providers vs not respectful professional HCWs). FGD participants also preferred kind and welcoming HCWs who were attentive to patient privacy and confidentiality.

*We expect that when we come here for a service that we be treated like normal people in the same way that someone with flu or a headache is treated and not to be labelled as ‘the ones who have come for medication’*….*–* 39 year-old man (FGD)

#### Efficiency: Less frequent visits and shorter wait times

DCE participants preferred less frequent appointments and shorter waiting times. They did not differentiate between a wait of 30 *vs*. 60 minutes but preferred 60 minutes to 2 hours (OR 1.15, CI 1.02–1.31) or 4 hours (OR 1.21; CI 1.04–1.40). Visits every six months were preferred to visits every three months (OR 1.092; CI 1.02–1.17). Participants had no preferences related to HF operating times, including the availability of morning, evening or weekend operating hours, or for HF closer to *vs*. further from their homes. There were only minor differences in these preferences between male *vs*. female and older *vs*. younger patients and data from the interaction analyses are not presented in this paper. Although the study was not designed specifically to assess the difference in preferences based on general health, we found that for participants who reported being bothered by symptoms related to their HIV status, longer visit spacing was not a significant driver of choice, while asymptomatic participants had a significant preference for six-month over three-month visit spacing (OR 1.162; CI 1.06–1.28, data not shown in the figure).

When asked which models they would be likely to join, more survey participants said they would be “very likely” to join a Fast Track model than any other. Participants reporting physical problems related to HIV infection were less likely to express interest in this model (OR 0.76; CI: 0.60–0.98) compared to those with no HIV-related symptoms (see Fig 2). There were no other significant differences in preferences between younger and older patients, between men and women, or between participants who reported symptoms of depression and/or physical symptoms that they associated with HIV and those who did not report such symptoms. Data from these interaction models are presented in more detail elsewhere [19].

FGD participants also reported strong preferences for less frequent visits, highlighting this as one of their top two priorities. They felt visits would ideally take place once or twice a year with larger supplies of ART dispensed at each visit and shorter/more efficient clinic visits. Unlike the DCE participants, they also valued more flexible clinic hours; the results of the DCE suggest that this was less significant in its importance in relation to other service delivery characteristics.

KII participants concurred with the need for efficiency. Seventeen of the 35 HCWs (49%) chose Fast Track as their favorite model and 7/35 (20%) preferred the Family Pick-up model; the rationale for both choices is that these reduce both facility congestion and HCW workloads. When asked which models they thought patients preferred, 23/35 identified Fast Track, which they felt patients appreciated because of its privacy and efficiency.

#### Location: Health facility-based services

As illustrated in Fig 2, survey participants were more likely to select facility-based over community-based DART models when asked how likely they would be to join. DCE participants also preferred most HF-based services. HFs close to home (reference category) were preferred to home-based services (OR 0.74; CI 0.64–0.86) and community-based services (OR 0.74; CI 0.71–0.92). When a distant HF was used as a reference category (not shown in the figure), participants preferred distant HF to home-based services (OR 1.26; CI 1.10–1.43) but had no preference between distant HF and community-based services.

FGD participants noted that service location was one of their top two priorities, and preferred clinic-based services to home-based or community-based models. Participants were worried about other community members finding out that they were on ART if services were provided at home or in the community.

*…Everyone goes to the clinic so no one will know why you are coming here* [to the clinic], *you may say you have a headache…Home delivery is a no*…– *25* year old woman (FGD)

*… I don’t like them* [medications] *to be brought home because my neighbors may know and I may lose hope*. *I will lose hope forever*.*–* 43 year old woman (FGD)

Some participants were concerned that they would not receive comprehensive services, such as psychosocial support, if services were provided at home or in the community:

*…getting treated at the clinic is important…because there are times when you will be at the clinic*, *you can discuss until you are satisfied… than at home or at shops where you can just go and pick your pills there is no time for discussions*.– 42 year old woman (FGD)

KII participants preferred facility-based models with less-frequent appointments (e.g., appoinitment spacing, fast track visits and family ART pickups) and felt that patients did as well because they decongested facilities, reduced HCW workloads and offered greater flexibility to clients. CARGs were their second-most preferred model, selected by 14/35 (40%) HCWs for their ability to decongest busy HF. Eleven participants (31%) felt it was also preferred by patients due to its added convenience and access to psychosocial support.

#### Confidentiality: Individual DART models

Survey participants were most likely to select an individual DART model, with 72% of those asked whether they would join Fast Track saying they would be “very likely” to do so. Group models were less preferred, with 54%, 36% and 26% of participants reporting that they would be “very likely” to join the family group model, refill clubs or CARGs, respectively. DCE participants also expressed significant preferences for individual *vs*. group models (OR 1.3; CI 1.22–1.41).

FGD participants were also overwhelmingly opposed to group models. They preferred to be attended to alone or in the presence of family members of their choosing. The main reason given was concern for privacy.

*I think generally people want privacy*. *No one wants their health status to be known so people want to be seen individually and I think there is no one who put a sticker there*.—45 year old man (FGD)

*People are a problem*, *I want to come alone but I do not mind coming with a family member so that they know where I get my medication just in case I get sick*.—29 year old woman (FGD)

#### Cost

As described above, some FGD participants identified the cost of transportation to HFs and user fees as a negative element of their current treatment. DCE participants had no preference between services costing US$1 and free services but increasing cost from US$1 to US$3 decreased acceptability (OR for $3 *vs*. $1 = 0.763; p<0.001), as did increasing cost to US$10 (OR for $10 *vs*. $1 = 0.323; p<0.001). FGD participants expressed mixed views, with some recommending free services altogether and others explaining that they would pay whatever amount as long as the medication was available.

## Discussion

The results of the KIIs, FGDs, survey and DCE were largely consistent. The preferences of stable, ART-experienced adult patients at these urban HIV treatment sites most often aligned with the characteristics of facility-based individual models, such as appointment spacing with multi-month prescribing and fast-track visits, rather than group or community-based models. These findings are consistent with the results of a recent DCE in Zambia [20]. The qualitative data showed that convenience, efficiency and privacy outweighed concerns about the cost of HF-based services and the potential advantages of group models, such as peer support. The DCE results highlighted that the most important variable for participants was provider attitude, a finding that is consistent with other studies [21, 22, 23, 24].

The study findings are also consistent with the lower uptake of community-based group models such as CARGs in Zimbabwe’s cities. The proximity of most participants to their treating HF seen in the study sample is characteristic of many urban settings, and stands in contrast to rural areas, in which people living with HIV often travel significant distances to reach HFs [25, 26]. In this study, the ease with which participants accessed their ART clinics reduced the appeal of community-based services. Although social networks in cities are often quite different from those in rural or peri-urban settings [27, 28], the majority of participants had family, trusted friends and support systems, and almost all had disclosed their HIV status to at least one person, countering the stereotype of urban isolation.

Strengths of this study include its use of qualitative and quantitative methods to triangulate data collection, inclusion of the perspectives of both HCWs and patients, and the rigor of the DCE approach. While the sample size was robust, participants were not randomly selected, and therefore may not be representative of all urban ART patients. The study also did not compare the preferences of urban and rural ART patients. In addition, participants described their preferences for hypothetical attributes and models that they had not yet experienced. It is possible that some concerns—for example, about confidentiality associated with group models—would be lessened once individuals actually participated in those models, as has been reported in a study from Zambia [29].

## Conclusions

These results are highly policy-relevant for Zimbabwe, suggesting the need to expand access to facility-based individual models in urban settings. By prioritizing the scale-up of appointment spacing with multi-month prescriptions and Fast Track visits, in addition to empowering individual HFs to choose additional contextually-appropriate DART models, MoHCC can meet the needs and expectations of HIV-positive patients while also creating efficiencies within the health system.
